# VA’S EXPERIENCE IN BUILDING CAPABILITY TO CONDUCT OUTREACH TO VULNERABLE PATIENTS DURING EMERGENCIES

**DOI:** 10.1093/geroni/igad104.0690

**Published:** 2023-12-21

**Authors:** Tamar Wyte-Lake, Pete Brewster, Terrence Hubert, June Gin, Darlene Davis, Chad Holmes, Aram Dobalian

**Affiliations:** U.S. Department of Veterans Affairs, North Hills, California, United States; VHA Office of Emergency Management, Martinsburg, West Virginia, United States; VHA Office of Healthcare Transformation, Washington, District of Columbia, United States; Veterans Emergency Management Evaluation Center, North Hills, California, United States; Geriatrics and Extended Care, Veterans Health Administration, Tampa, Florida, United States; VA Service and Support Center, Denver, Colorado, United States; Veterans Emergency management Evaluation Center, North Hills, California, United States

## Abstract

The United States (US) has sustained more than 340 weather and climate disasters since 1980. Older adults with complex medical conditions, increasing in numbers, are typically most severely impacted by such events. A growing population of older, medically complex, individuals residing at home, and an increased frequency of severe natural disasters creates a compounded burden on local communities to reduce potential harm to these community-dwelling members during disaster response and recovery. In response, the Veterans Health Administration (VHA) established the Vulnerable Patient Care, Access, and Response in Emergencies (VP CARE) program to provide standardized data tools and guidance to assist VA medical facilities in conducting outreach to and care coordination of medically frail and older Veterans during major emergencies. VP CARE utilizes geographic information system (GIS) tools to allow outreach prioritization based upon geographic proximity to an event and patient clinical needs. In response to Hurricanes Ian and Fiona, the VP CARE team, with the support of the national VEText team, successfully deployed a new 2-way patient texting communication capability. During an emergency, patients receive a text from VEText, are asked whether they require assistance, and the specific help they need. The new VEText capability helped VA staff reduce outreach call volume by 20% and allowed for triage and prioritization of those indicated as needing assistance to the top of the outreach list. This presentation provides an overview of VP CARE approaches and what VHA has learned through its implementation to assist other healthcare systems with establishing similar capabilities.

